# Rural-urban differentials in early childhood education and child development: Evidence from Multiple Indicator Cluster Survey (MICS) in Ghana

**DOI:** 10.1371/journal.pgph.0002171

**Published:** 2023-08-07

**Authors:** Martin Wiredu Agyekum, Sylvia Boamah Yeboah, Charity Dzradosi, Kingsley Ofosu-Ampong, Michael Odenkey Quaye, Christie Donkoh, Andrews Acquah, Cosmos Kwame Dzikunu, Edison Pajibo, Daniel Yelkpieri, Emmanuel M. J. Tamanja, Ephraim Avea Nsoh

**Affiliations:** 1 Institute for Educational Research and Innovation Studies, University of Education, Winneba, Mampong, Ghana; 2 Faculty of Human and Social Studies, Mykolas Romeris University, Vilnius, Lithuania; 3 Department of Adult Education, University of Ghana, Legon, Accra, Ghana; 4 Department of Information Technology, University of Professional Studies, Accra, Ghana; 5 Department of Agricultural Science Education, University of Education, Winneba, Mampong, Ghana; Jawaharlal Nehru Medical College, INDIA

## Abstract

Children’s early development is a key component that affects their wellbeing and health as they age. In recent times, scholars’ interest in Early Childhood Education (ECE) and Early Child Development (ECD) has grown exponentially. However, rural-urban differentials in early childhood development in sub-Saharan Africa (SSA) and particularly in Ghana are unknown. This study examined the rural-urban differentials in Early Childhood Education (ECE) and Early Child Development (ECD) in Ghana. We used cross-sectional data from 2017/2018 Multiple Indicator Cluster Survey (MICS) by the Ghana Statistical Service. We restricted the data to children aged 3 and 4 years. A sample size of 3683 children was used in this study. Poisson regression analysis was used to examine the relationship between Early Childhood Education (ECE) and Early Child Development (ECD) in rural and urban Ghana. Both ECE and ECD scores were higher in the urban areas than in the rural areas. The regression shows the rural-urban disparity in ECD by ECE. The Preschool Program (ECE) has a good impact on children’s early development in Ghana. However, the Relative Risk Ratio (RRR) in rural areas was higher than in urban areas. Beyond this, other factors such as age of child, ecological zone, maternal education and wealth index were associated with ECD. Our findings show a significant disparity in Early Childhood Education and Childhood Development in rural and urban areas This study therefore recommends that more resources be channeled in rural areas to help improve ECE and ECD while policies should be tailored to Early Childhood Education.

## Introduction

Early Child Development (ECD) is a multidimensional concept that comprises children’s physical, cognitive, social and emotional development [1–4]. A child’s development starts from conception through to the early years after birth. This enables the child to build the capacity for healthy life, resulting in good well-being [1]. Early Childhood Development has gained national and international recognition due to its health and social impact such as poor child growth and impaired cognitive development [5, 6]. For instance, the United Nations Sustainable Development Goals (SDG) emphasize the importance of Early Childhood Development. Specifically, Target 4.2 of SDG 1 states that by 2030, “countries should ensure that all girls and boys have access to quality early childhood development, care and pre-primary education so that they are ready for primary education” [1]. Although early childhood development is very essential for children’s health and well-being in later life, the development of children may be hindered in most sub-Saharan African countries, especially in the rural areas where access to resources for children’s development is limited [6]. Britto et al. [7], argued that approximately 20% of children aged less than 5 years develop slowly. This is attributed to protection and nurturing care from parents, family and community factors which consequently affect the health and well-being of children, thereby impacting on their ability to learn [7]. In addition, it is estimated that about 250 million children, approximately 43% in low and middle-income countries are at the risk of not reaching their development potential [3].

Evidence from literature shows that there is a global disparity in early childhood development and several factors such as socio-demographic, economic, and early childhood education have been identified to have an influence on early childhood development [8–10]. Early Childhood Education (ECE) is very significant in early childhood development. This mostly occurs in children’s formative years when they start developing cognitive and non-cognitive skills through ECE programs [11, 12]. Through ECE programmes, children are able to develop their learning skills, physical and mental health outcomes. Hence, high-quality pre-school is very important for the development of children’s cognitive and socio-emotional development [1, 9]. Investment in ECE is a way to promote children’s development, learning, school readiness, and reducing long-term poverty as well as improving the future labor force [13]. In sub-Saharan Africa, about 17.9% of children aged three (3) and four (4) years old and 61 .7% of children in Latin America and the Caribbean have access to ECE [14].

In Ghana, much emphasis has been placed on ECE due to its impact on ECD. Ghana launched it’s ECE policy in 2004 to improve access to quality kindergarten education. In 2007, the policy and curriculum were revised to include two (2) years of pre-primary education and making it compulsory for all children [13]. The curriculum aims to improve children’s development, enable them to develop good communication skills, familiarize with the environment and develop psycho-social competencies and creative skills [15, 16]. Currently, Ghana has abolished fee for basic education in all public schools, students in both rural and urban areas do not pay school fees. Aside this, the government of Ghana has introduced the School Feeding Programme (SFP) where children in primary schools in poor and deprived communities are fed once a day [11, 16]. Despite these policy frameworks, early child development remains a significant problem in the educational sector due to differences in the availability of resources in both urban and rural areas though there has been effort by the government to ensure equitable distribution of resources [17]. Evidence shows that place of residence (rural or urban) plays a critical role in child development [11].

Several studies have been conducted on the relationship between Early Childhood Education and Early Child Development in developed countries and sub-Saharan Africa [6, 17, 18] but few of such studies in Ghana [11, 19]. Evidence shows that there is disparity in ECE with regard to the place of residence and its impact on ECD [1, 11]. However, the few studies on ECE and ECD have not examined the rural-urban differences. In Ghana, the difference in place of residence is very important towards child education and development. This is due to the availability of resources and pre-schools in both urban and rural areas where access to education is higher in urban than rural areas [20]. Most preschools in rural areas are underprivileged compared to urban preschools [20, 21]. Given that studies on Early Childhood Education and Development were conducted at the individual country level, there is no study on the rural-urban disparities. This suggests a significant knowledge gap in the current discourse of rural-urban differentials in Early Childhood Education and Early Child Development in Ghana. An understanding of the rural-urban disparities could help in designing and implementing specific strategies that can improve Early Child Development to help Ghana achieve SDG 1 target 4.2. Therefore, this study examined the rural-urban differentials in Early Childhood Education (ECE) and Early Child Development (ECD) in Ghana.

## Materials and methods

### Study design

This study used data from the 2017/2018 Multiple Indicator Cluster Survey (MICS) by the Ghana Statistical Service. The MICS is a national representative cross-sectional survey that uses a multi-stage stratified cluster sampling approach. The financial and technical support of MICS is supported by international institutions such as the United Nations Children’s Fund (UNICEF), United State Agency for International Development (USAID), Korean International Cooperation Agency (KOICA), United Nation Development Programme (UNDP) and the World Bank through the statistics for Results Facility–Catalytic Fund (SRF-CF). The first stage involved the selection of clusters consisting of Enumeration Areas (EAs) in the rural-urban areas. This was followed by the systematic sampling of households and listing of households in the selected clusters in the enumeration areas. The survey collected data from households, women, men, children aged 5–17 years and children less than 5 years [22]. For the purpose of this study, the children and women files were merged. Hence, the study was restricted to children aged 3 and 4 years. A sample size of 3683 children was used in this study. The dataset is publicly available and can be obtained with written permission from https://www.statsghana.gov.gh/gssdatadownloadspage.php. Verbal consent was obtained from each respondent. Participation in the survey was completely voluntary. Participants were recruited from August to September, 2017. All datasets were aggregated after they were completely anonymized and de-identified. Therefore, we are unable to link the identities of any respondent to the data. The survey protocols were approved by the Ghana Health Service Institutional Review Board.

### Measurement of variables

#### Dependent variable

The dependent variable for this study was the Early Childhood Development Index (ECDI) of children aged 36–59 months. McCoy argued that ECD items are very appropriate for children 3–4 years as they reflect the general behaviour, emotions, and skills for children within that age period. In addition, MICS assesses ECD among children aged 36–59 months [23]. This was computed from 10 items from 4 domains such as physical, literacy, social emotions and learning. The physical domain had 2 items and they were (1) if the child can pick up a small object with two fingers, like a stick or a rock from the ground (2) if the mother/caretaker does not indicate that the child is sometimes too sick to play. The literacy domain had 3 items, which included (3) whether the child can identify/name at least ten letters of the alphabet? (4) whether the child can read at least four simple, popular words? (5) whether the child knows the name and recognizes the symbols of all numbers from 1 to 10? Social emotions had 3 items and these included (6) if the child gets along well with other children, (7) if the child does not kick, bite, or hit other children, (8) if the child does not get distracted easily. Lastly, the learning domain had 2 items (9) if the child follows simple directions on how to do something correctly, (10) when given something to do, is able to do it independently. The responses for each of the items were “yes” and “no”. The Yes responses were recorded as “1” and the No responses were recorded as “0”. The Early Childhood Development Index was computed by summing all the 10 items.

#### Independent variable

The independent variable for the study is Early Childhood Education. During the survey, respondents were asked if children aged 3 and 4 years have ever attended an Early Childhood Education (ECD) program. Those who responded that they have ever attended ECD programs were classified as “Yes” whiles those who responded that they have never attended ECD programs were classified as “No”.

#### Control variables

The control variables for the study are sex, age of a child, the child reads at least four simple popular words, disability of child, age of mother, place of residence, mother’s education, marital status, wealth quintile and region of residence. These variables were selected based on the literature [1, 3, 8, 11, 21]. Sex of the child was categorized as males and females, the age of a child was classified as 3 years and 4 years. In addition, the child reads at least four simple popular words was categorized as “Yes” and “No”. Disability of children was categorized as “functional difficulty” and “no functional difficulty” according to UNICEF child functional module classification [23, 24].

In addition, the age of the mother was classified as 15–19 years, 20–24 years, 25–29 years, 30–34 years, 35–39 years, 40–44 years and 45–49 years. Place of residence was categorized as rural and urban. The highest level of education of mothers was coded as no education, primary, Junior High School (JHS/Middle), secondary and tertiary. Marital status was categorized as currently married, never married and ever married. Moreover, the household wealth quintile was categorized as poor, poorer, middle, rich and richer. For the region of residence, the data was collected using the ten (10) administrative regions (Greater Accra, Volta, Central, Western, Eastern, Ashanti, Brong Ahafo, Northern, Upper East and Upper West) in Ghana. This was recategorized as Southern (Greater Accra, Volta, Central, Western), Middle (Eastern, Ashanti, Brong Ahafo) and Northern Zones (Northern, Upper East and Upper West).

### Data analysis

The data for the study was analyzed using STATA version 16. The proportion of the variables were examined using percentages and frequencies. The results were further disaggregated by place of residence. Multivariable Poisson Regression analysis was carried out to explore the predictors of Early Childhood Development index by place of residence. Poisson regressions were run because the dependent variable Early Childhood Development index was a count variable. Three poisson regressions were run. The first model examined the factors associated with Early Childhood Development index using the pooled data. The second and third model examined the urban-rural differentials in factors associated with Early Childhood Development Index. The Relative Risk was used to explain the significant variables.

### Ethical approval

Ghana Statistical Service received the ethical approval to conduct this study before the data was collected. Verbal consent was obtained from each respondent participating and, children age 15–17 years were individually interviewed after adult consent had been obtained in advance from their parents or caretakers.

## Results

The Early Childhood Development Index (ECDI) and background characteristics of the children are presented in Table 1. Generally, the result of our analysis shows that the average Early Childhood Development Index was 5.91. Further examination of the result shows that about 68.67% of the children were currently attending Early Childhood Education programs during the survey. With regard to sex, more than half (50.69%) of the children were females, and slightly less than half (48.98%) of the children were 4 years. Also, about eight out of the ten (82.41%) children could read at least four simple popular words. With regards to child functional difficulties or disability, less than one-tenth (8.47%) had functional difficulty in performing any activity.

**Table 1:** Background characteristics of the children.

**Measure**	**Mean (std. deviation)**	**Min, Max**
**Early Childhood Development Index**	5.91 (1.77)	0.10
**Urban**	*6*.*50 (1*.*73)*	
**Rural**	*5*.*52 (1*.*69)*	
**Children’s characteristics**	**Frequency**	**Percentage**
**Currently attending Early Childhood Education Program**		
No	1,154	31.33
Yes	2,529	68.67
**Sex**		
Male	1,816	49.31
Female	1,867	50.69
**Ages of children**		
3years	1,879	51.02
4years	1,804	48.98
**The child reads at least four simple, popular words**		
Yes	648	17.59
No	3,035	82.41
**Disability status**		
Functional difficulty	312	8.47
No functional difficulty	3.371	91.54
**Total**	**3683**	**100**

Table 2 shows the maternal characteristics. The results show that one-fifth (20.17%) of the mothers were between 30–34 years, 17.32% were 35–39 years, one-tenth (11.13%) were 40-44years and the least proportion (6.46%) were between 45–49 years. With regard to education, more than one-third (37.52%) of the mothers had no education and the least proportion (4.59%) had tertiary. Also, more than two-thirds (87.05%) of the mothers were currently married and 5.95% were never married. In terms of household wealth, 30.87% of mothers belonged to the poorest wealth quintile, and slightly less than one-fifth (18.54%) of the women belonged to the middle household wealth quintile. More than one-third (36.08%) of the women resided in the southern sector and 31.14% were in the middle zone. In addition, 6 out of 10 women were living in the rural areas.

**Table 2:** Background characteristics of mother.

Mother’s Characteristics	Frequency	Percentage
**Age of mother**		
15–19	501	13.6
20–24	470	12.76
25–29	683	18.54
30–34	743	20.17
35–39	638	17.32
40–44	410	11.13
45–49	238	6.46
**Mother’s Education**		
No education	1,382	37.52
Primary	693	18.82
JSS/JHS/Middle	1,138	30.90
SSS/SHS/ Secondary	301	8.17
Higher	169	4.59
**Marital status**		
Currently married	3,206	87.05
Formerly married	258	7.01
Never married	219	5.95
**Wealth quintile**		
Poorest	1,137	30.87
Poor	718	19.49
Middle	683	18.54
Rich	585	15.88
Richest	560	15.2
**Region of residence**		
Southern	1,329	36.08
Middle	1,147	31.14
Northern	1,207	32.77
**Place of residence**		
Urban	1,473	39.99
Rural	2,210	60.01
**Total**	**3683**	**100**

### Factors associated with Early Childhood Development Index

From Table 3, the results of the poisson regression show that children attending early childhood education, age of the child, ecological zones, mothers’ educational level and household wealth index were associated with Early Childhood Development Index.

**Table 3:** Factors associated with early childhood development in Ghana.

Early Childhood Development Index	Pooled data		Urban		Rural	
	IRR (95% CI)	p-value	IRR (95% CI)	p-value	IRR (95% CI)	p-value
**Currently attending ECE**						
No (RC)						
Yes	1.14 (1.10–1.17)	**0.000**	1.13 (1.07–1.20)	**0.000**	1.14 (1.09–1.18)	**0.000**
**Place of residence**						
Urban (RC)						
Rural	0.96 (0.93–0.99)	**0.016**				
**Sex**						
Male (RC)						
Female	0.99 (0.97–1.02)	0.580	0.99 (0.95–1.03)	0.569	1.00 (0.96–1.03)	0.886
**Age of child**						
3 years (RC)						
4 years	1.08 (1.05–1.11)	**0.000**	1.11 (1.06–1.15)	**0.000**	1.06 (1.02–1.10)	**0.003**
**Ecological zones**						
Southern (RC)						
Middle	1.02 (0.99–1.06)	0.157	1.01 (0.96–1.05)	0.733	1.04 (1.00–1.09)	0.0.69
Northern	0.94 (0.91–0.98)	**0.004**	0.94 (0.89–1.00)	0.068	0.96 (0.91–1.01)	0.101
**Age of woman**						
15–19 (RC)						
20–24	0.99 (0.94–1.05)	0.936	1.02 (0.93–1.12)	0.675	0.98 (0.92–1.06)	0.676
25–29	0.99 (0.95–1.05)	0.915	1.01 (0.94–1.09)	0.791	0.99 (0.93–1.05)	0.739
30–34	1.01 (0.96–1.06)	0.629	1.03 (0.96–1.11)	0.377	1.01 (0.94–1.07)	0.986
35–39	1.01 (0.96–1.06)	0.712	1.03 (0.95–1.11)	0.482	0.99 (0.94–1.06)	0.949
40–44	1.02 (0.96–1.07)	0.615	1.05 (0.97–1.14)	0.228	0.99 (0.92–1.06)	0.748
45–49	1.03 (0.97–1.10)	0.316	1.09 (0.99–1.21)	0.081	1.01 (0.92–1.09)	0.959
**Educational level of mother**						
No education (RC)						
Primary	1.02 (0.98–1.06)	0.442	1.02 (0.95–1.09)	0.591	1.01 (0.96–1.06)	0.743
JSS/JHS/Middle	1.06 (1.02–1.10)	**0.002**	1.03 (0.97–1.10)	0.287	1.08 (1.03–1.14)	**0.002**
SSS/SHS/Secondary	1.10 (1.04–1.16)	**0.001**	1.08 (1.01–1.17)	**0.029**	1.09 (0.99–1.20)	0.077
Higher	1.06 (0.98–1.13)	0.155	1.03 (0.95–1.12)	0.481	1.08 (0.92–1.27)	0.336
**Marital status**						
Currently married (RC)						
Formerly married	1.02 (0.96–1.07)	0.632	1.00 (0.93–1.07)	0.936	1.03 (0.95–1.11)	0.513
Never married	1.03 (0.98–1.09)	0.352	1.02 (0.94–1.10)	0.655	1.04 (0.95–1.13)	0.361
**Wealth index**						
Poor (RC)						
Middle	1.07 (1.02–1.11)	**0.002**	1.02 (0.95–1.09)	0.584	1.09 (1.04–1.15)	**0.001**
Richest	1.12 (1.07–1.16)	**0.000**	1.09 (1.03–1.17)	**0.005**	1.11 (1.04–1.17)	**0.001**
**Functional Disability**						
Functional difficulty (RC)						
No functional difficulty	1.01 (0.95–1.05)	**0.963**	1.04 (0.96–1.12)	**0.387**	0.98 (0.92–1.04)	**0.520**

Children who were currently attending Early Childhood Education programs had a higher (IRR = 1.14; 95%CI = 1.10–1.17) Early Childhood Development Index than those not currently attending Early Childhood Education programs. Likewise, children who are currently attending Early Childhood Education programs in both urban (IRR = 1.13; 95%CI = 1.07–1.20) and rural (IRR = 1.14 (1.09–1.18)) areas had a higher Early Childhood Development Index than those not currently on Early Childhood Education programs.

Children aged 4 years had higher Early Childhood Development Index than those aged 3 years. Similar association was observed in the rural and urban areas.

On ecological zones, children residing in the northern zone had a lower (IRR = 0.94; 95% CI = 0.91–0.98) Early Childhood Development Index than those residing in the southern zone. For the rural-urban differentials, no significant association was observed.

Education of mother was associated with Early Childhood Development. Children of mothers with Junior High school had a higher (IRR = 1.06; 95%CI = 1.02–1.10) Early Childhood Development compared to those with no education. Also, children of mothers with Senior High School education had a higher (IRR = 1.10; 95%CI = 1.04–1.16) Early Childhood Development compared to those with no education. With regards to the rural-urban differentials, there were slight differences. In rural areas, children of mothers with Senior High School education had a higher (IRR = 1.08; 95%CI = 1.01–1.17) Early Childhood Development compared to those with no education. For the rural areas, children of mothers with Junior High school had higher (IRR = 1.08; 95%CI = 1.03–1.14) Early Childhood Development Index compared to those with no education.

With regards to household wealth index, children of mothers who belonged to middle households (IRR = 1.07; 95% CI = 1.02–1.11) and richest households (IRR = 1.12 95% CI = 1.07–1.16) had a higher Early Childhood Development Index compared to those belonging to the poor households. For the rural urban differentials, similar direction and association were observed for rural residence as in the pool data. However, in the urban areas, only rich household was associated with Early Childhood Development Index. Thus, children of mothers belonging to the household wealth quintile had higher (IRR = 1.09; 95% CI = 1.03–1.17) Early Childhood Development Index compared to those belonging to those in poor households.

## Discussion

Previous studies have highlighted the difference in place of residence with regard to Early Childhood Education and Early Child Development in Ghana and sub-Saharan Africa. However, there is no evidence of the factors associated with Early Child Development in rural and urban areas in Ghana. Drawing on data from a national representative survey, thus, the 2017/18 Multiple Indicator Cluster Survey (MICS), this study examines the rural-urban differentials of the relationship between Early Childhood Education and Early Childhood Development. The study contributes to Early Childhood Education and Early Childhood Development to help improve child development and help to achieve Sustainable Development Goal 1 in Ghana.

The finding of the study points to the rural-urban differentials in ECE and ECD. The results show that the mean Early Child Development score was 5.91 with a standard deviation of 1.77. With a minimum and maximum score of 1 and 10 respectively, the mean score of 5.91 could be interpreted as 59.1%. When compared with other countries, the ECD index in this study is lower than findings from other studies in Nepal and Bangladesh [25, 26]. This could be attributed to time of data collection, socio-economic difference and country settings.

We found that there was a difference in the rural-urban ECD score. The mean score was higher in urban areas (Mean = 6.50, std deviation = 1.73) than the rural areas (Mean = 5.52, std deviation = 1.70). The results imply that the mean score for the urban area is higher than the general score for Ghana. The significant difference in the mean score for Early Child Development in rural and urban areas necessitates the factors that account for Early Childhood Development in rural and urban Ghana. This result was expected because there are more resources in the urban areas than in the rural areas that aid in the development of children [20, 21]. Similarly, for Early Childhood Education, the pooled data showed that 68.67% of children during the survey were currently enrolled in Early Childhood Education. The rural-urban differentials showed that ECE was higher in the urban areas (82.55%) than in the rural areas (59.41%). This finding, therefore, underscores the need to improve Early Childhood Education in rural areas.

The regression shows the disparity in rural-urban in ECD by ECE. Preschool program has a good impact on a child’s early development in Ghana. However, the IRR in rural areas was higher than in urban areas. The data stresses a need for a qualitative study to explain the regression results. This is corroborated in research by Bago et al. [11] who reported that a relationship between Early Childhood Education and Early Childhood Development. Children who were officially enrolled in Early Childhood Education programs had a higher Early Childhood Development Index than those who were not. This was also evident in both rural and urban areas where children who were enrolled in Early Childhood Education programs had a higher Early Childhood Development Index than those who are not. This shows that in whatever area a child is, ECE is very important in his or her development. This result is generally in line with earlier studies on early education, which implies that ECE has a beneficial impact on young students’ overall academic progress. The outcomes of children’s early development should be positively impacted in the long run by investments in early education [18]. This implies that policy initiatives to support ECE are required, particularly for the most disadvantaged and poor children, in order to enhance child literacy, physiological, and social wellbeing in Ghana. One of such key policy is the development of ECE Policy Framework building. The framework ensures that there are more spaces for kindergarten, effective implementation of KG curriculum for improved play-based learning and enhancing social change through the reduction of inequality amongst the vulnerable and marginalized children. This policy takes into consideration students in both rural and urban areas, and therefore seeks to bridge the gap between rural and urban areas. Consequently, this could account for less differences in the rural and urban disparities found in this study.

We also found rural-urban disparities in Early Childhood Development by the age of the child. For the pooled data, the regression results show that the relative risk of ECD was higher for children aged 4 years than those aged 3 years. This implies that the IRR for ECD increases with an increase in age. The result is in agreement with a study by Hag et al., [1] in Bangladesh, Costa Rica and Ghana who reported a significant positive relationship between age of child and ECD. They indicated that children aged 4 years have higher ECD than those aged 3 years. The reason could be attributed to the development of children. Childhood development occurs as children grow. Socioemotional development occurs at age one where attachment formation becomes very critical for children’s growth. As the child grows, emotional skills and other developmental traits such as identification, start to develop. Therefore, these factors could account for children aged 4 years than 3 years having higher cognitive development.

Ecological zones also emerged as a significant factor in ECD only for the pool data. This could be attributed to the low development and lack of resources in the northern part of Ghana. There are less availability of resources or educational facilities aiding in ECD and ECE in the northern part than the Southern zone of Ghana [27], and this can have an influence on a child’s early development. The availability of resources could facilitate child learning and thereby help in Early Childhood Development. In that, children in the Southern zone may have access to learning resources compared to children in the Northern zone. This could therefore account for the differences in the results of Early Childhood Development in this study. As a way of improving resources in the Northern Ghana, several efforts have been made by Non-Governmental Organizations and developing agencies to help improve accessibility, and Teaching and Learning Materials (TLMs) in the basic schools.

Our findings also show that maternal education is associated with ECD for both the pooled data and rural-urban disparity. The findings of this study are analogous to other studies [1, 28] which reported that formal education has an influence on Early Childhood Development. For the pooled data, both women with JSS/JHS/Middle and SSS/SHS/Secondary of children had higher ECD than women with no education. Maternal education has been identified as a major factor towards child development. Educated mothers are exposed to child development factors and would provide the means and ensure a favorable environment both at home and in school for child development. However, there was a difference with regard to the rural-urban disparity. In the urban areas, children of mothers with secondary education had a higher ECD than those without education. In the rural areas, children of mothers with JHS/Middle education had a higher ECD than those without education. The differences were expected in that, in the rural areas, most of the mothers were not educated and the few who were educated saw the relevance of education towards their child’s development [28]. The rural-urban findings show that regardless of the place of residence, educated mothers could provide resources or environment for their children to develop. In line with this, it becomes prudent to implement and improve upon policies that focus on parent education and involvement in child care at home since it is a crucial addition to enhancing children’s academic performance.

Wealth index was identified as a significant factor towards child development. Children from middle or rich families had higher ECD than those from poor families. This was similar to the rural-urban disparities. The study’s findings are similar to other studies [3, 29]. The probable reason could be that children from wealthy families may have the advantage of having or acquiring resources that would enable their children to grow. They are mostly at the better position to incur any cost to ensure the wellbeing of their children’s early development. According to Black et al. [3], wealth gap leads to difference in children’s cognitive development, which is portrayed in this study’s results. Also, the economic status of a family determines the food security of a child, his or her enrolment in ECE, amongst other beneficial factors that influences ECD. Lack of finances contributed to the parents’ inability to provide the needed resources a child needs for development. This is evident in Aurino et al.’s [30], study which demonstrates that, early childhood food insecurity and malnutrition, which are typically encountered by low-income and middle-class families, can have negative long-term and generational repercussions on health and education.

### Strength and limitation

This study used cross-sectional data and therefore acknowledges that it makes it very difficult to establish causal inferences between Early Childhood Education and Childhood Development. In addition, there could be a social desirability and recall biases. Some respondents may tend to give response which may not be the characteristics of their children as there is a tendency of respondents comparing their children with other children in the household or community. For instance, respondent may indicate that their child is emotionally or physically strong but the reality might be otherwise. In addition, there could be a situation where parents may not want to report that their children are not in school. The study was delimited to only variables available in the dataset. Other important variables relating to early childhood education and household environment which have potential impact on childhood development were not part of the study variables. Notwithstanding, the findings of the study is very relevant towards implementing policies that will aid in ensuring Early Childhood Education and Childhood Development.

## Conclusion

We found significant disparity in Early Childhood Education and Childhood Development in rural and urban areas. The mean score for both ECD and ECE was higher in urban areas than the rural areas in Ghana. The difference in the rural-urban Early Childhood Development is attributed to education of mother, ecological zones, age of mother and wealth index. Policies should target improving Early Childhood Education and Childhood Development. It is therefore recommended that more resources are channeled in rural areas to help improve ECE and ECD.
